# Misdiagnosed large bladder stone after laparoscopic radical prostatectomy and literature review: a case report

**DOI:** 10.3389/fonc.2025.1550520

**Published:** 2025-12-09

**Authors:** Wenbo Gao, Yangkai Xu

**Affiliations:** Department of Urology, Ningbo Urology and Nephrology Hospital, Ningbo, Zhejiang, China

**Keywords:** bladder stone, case report, prostate cancer, misdiagnosis, Hem-o-lok

## Abstract

Large bladder stone is an uncommon disease, and its diagnosis is sometimes challenging. Herein we reported the case of a 75-year-old male patient who was admitted to undergo open cystostomy for a diagnosis of “urinary incontinence, urethral stricture, post-radical prostatectomy state, and urinary tract infection”. However, during the operation, we found that he was misdiagnosed by preoperative MRI examination, and he had a large bladder stone measuring approximately 6 × 5 cm and weighing 105 grams. Furthermore, the stone was found to be caused by a migrated Hem-o-lok clip originating from the previous laparoscopic radical prostatectomy. The eventual treatment is open cystolithotomy. Postoperatively, careful re-interpretation of the MRI film revealed a stone in the bladder, but the large stone was missed preoperatively. Doctors should be aware of the characteristics of bladder stone on MRI examination, and the diagnosis of bladder stones formed by foreign bodies requires a combination of multiple imaging methods to be confirmed.

## Introduction

Bladder stones (BS) account for approximately 5% of all urinary stones (1). BS is an ancient disease with a very long history, and the oldest BS was found in the skeleton pelvis of a predynastic Egyptian boy, which was estimated to be more than 7,000 years ago (2). In the modern time, BS is especially more common in male adults than in female, particularly among those over the age of 45.

Patients with BS may develop multiple symptoms, for example, urinary tract infection (UTI), hematuria, increased urinary frequency, dysuria, and pain (3), which have serious outcomes on patients’ health and quality of life. BS is usually not very large as patients would seek medical advice when they present with such symptoms; consequently, a large BS is rare in clinical practice. Herein we reported a case with a large BS which was misdiagnosed initially; in particular, the BS was found to be caused by a migrated Hem-o-lok clip originating from the previous laparoscopic radical prostatectomy (LRP).

## Case description

A 75-year-old male patient referred to my clinic for complaints of increase urinary frequency, dribbling, feeling of incomplete voiding, mild lower abdomen pain, and urinary incontinence for approximately 1 year. About a week ago, at another tertiary hospital in our city, one urologist advised him to receive cystostomy as he had had a previous history of urethral rupture approximately 10 years before, with urethral stricture thereafter. The urologist had already tried urinary catheter insertion and cystoscopy examination, but both failed. The patient refused to undergo urethral strictureoplasty, so he asked me to perform cyststomy in our hospital as he heard that I had performed cystostomy for his friend with satisfactory result. Moreover, the patient had undergone LRP 4 years before for prostate cancer (PCa) without regular follow-up. Recently, his urine stream became much thinner, and more importantly, urine incontinence gradually occurred. Therefore, he decided to undergo cystostomy at our hospital. At admission, the physical examination was unremarkable except mild suprapubic tenderness while without rebound tenderness.

At that other tertiary hospital in our city, the patient underwent pelvic magnetic resonance image (MRI) examination because the urologist was concerned about his prior history of PCa, but the result showed “post-radical prostatectomy state, with consideration of neurological bladder and mild bilateral ureteral dilation. The bladder volume is smaller”. In our hospital, we read the pelvic MRI result but did not examine the MRI film very carefully as that tertiary hospital is also a large hospital, and we considered that their result was correct.

His hematologic and biochemical investigations were also unremarkable, with a serum creatinine level of 76 μmol/L and PSA value of 0.01 ng/mL. Urinalysis revealed microhematuria and pyuria (130 white blood cells and 65 red blood cells/high powered field), suggesting a urinary tract infection. His urine culture grew a pure growth of *Escherichia coli* that was susceptible to cefoperazone sodium and sulbactam sodium. A diagnosis of “urinary incontinence, urethral stricture, post-radical prostatectomy state, and urinary tract infection” was made. Given that the patient’s symptoms (urinary incontinence, frequency) and the external MRI report of a small-volume bladder were consistent with a neurogenic bladder secondary to radical prostatectomy and urethral stricture and considering the patient’s urgency to resolve the incontinence via cystostomy, we proceeded with the planned surgery without repeating imaging at our institution. Based on the report of urine culture and antibacterial sensitivity, after an intravenous injection of cefoperazone sodium and sulbactam sodium for 5 days, we scheduled to perform open cystostomy under general anesthesia.

**Figure 1:**
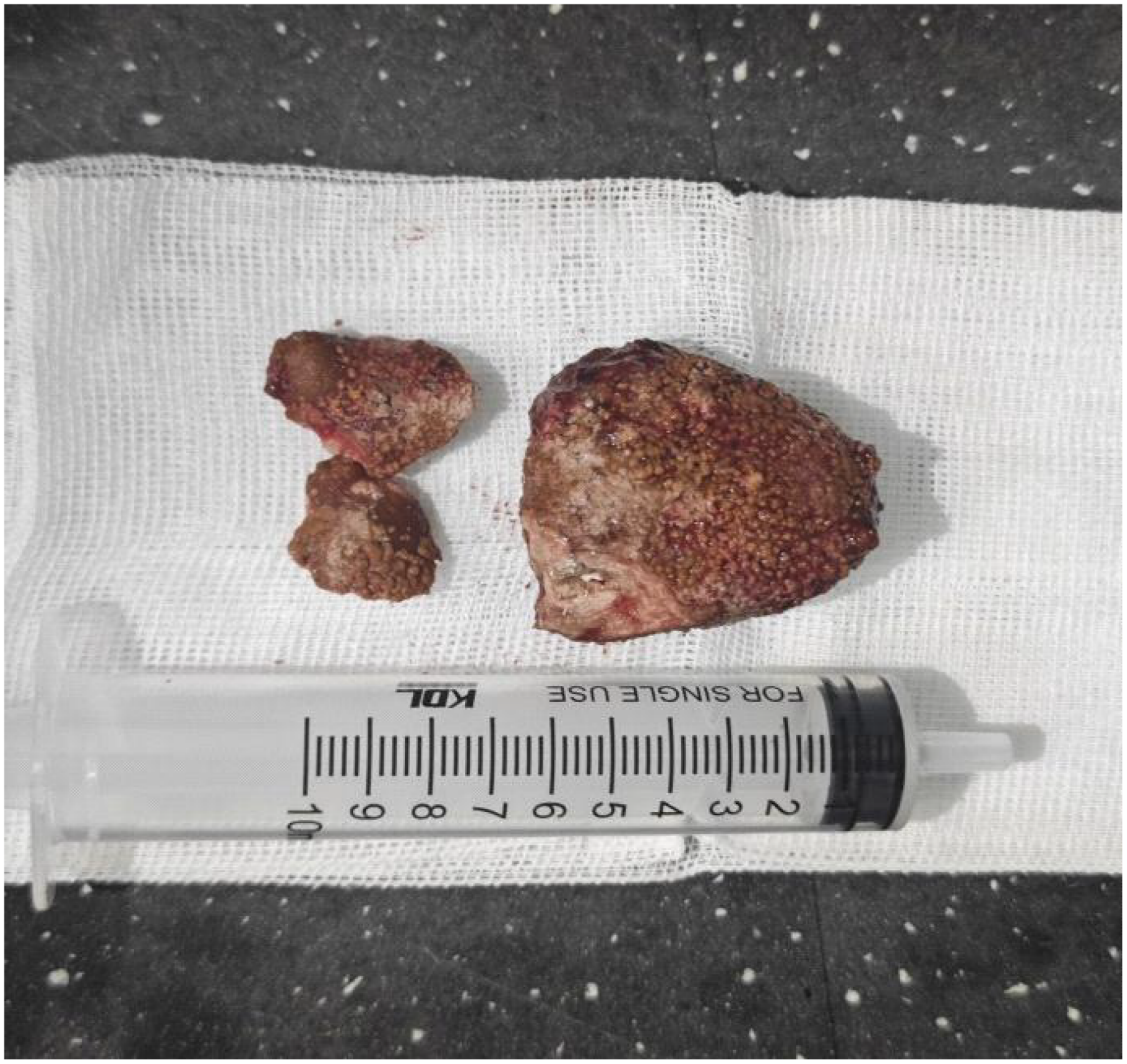
A large stone (approximately 6 × 5 cm) was extracted out of the bladder but broken into one bigger piece and two smaller ones.

**Figure 2:**
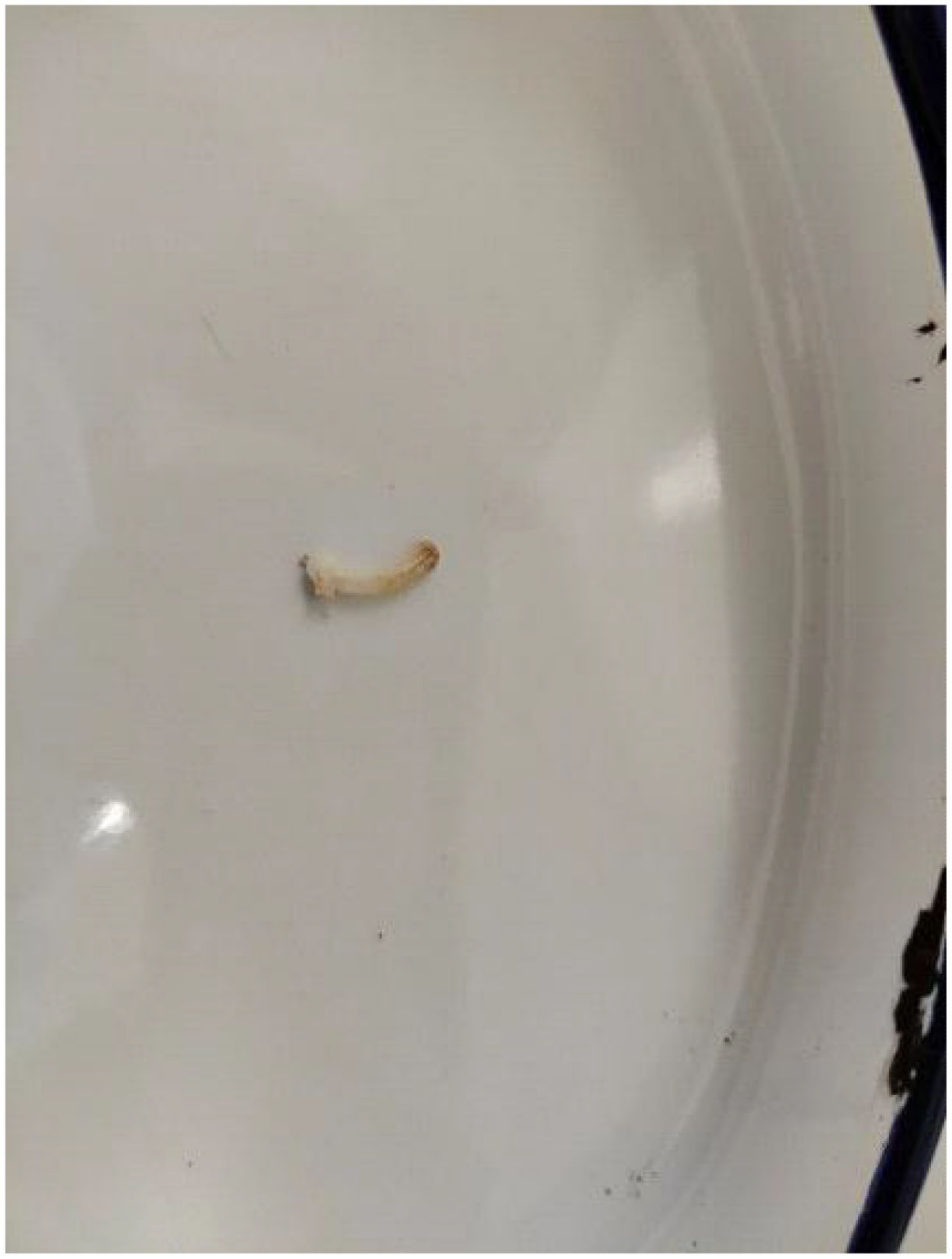
A Hem-o-lok clip was revealed after crushing the large BS.

During the operation, a 2-cm-long incision was made. Before insertion of the suprapubic tube into the bladder, I touched the inner surface of the bladder, but unexpectedly, a hard object was touched. Careful touching felt that it was a large stone. With a widened incision and aided by instruments, an oval stone with rough surface was extracted out, which measured approximately 6 × 5 cm and weighed 105 g. The bladder stone was mobile without adhering to the bladder, so no additional damage was rendered. Unfortunately, during the course of extraction, as the stone was too large and the incision of the bladder was small, it was broken into one bigger piece and two smaller ones, as shown in **Figure 1**. Following careful observation, no abnormal appearance of the bladder mucosa was visualized; thus, biopsy was not undertaken. Eventually, an open cystolithotomy was conducted. The surgery time was approximately 1.5 h, and blood loss was approximately 50 mL during surgery. When crushed, a closed white Hem-o-lok clip was found in the stone (**Figure 2**). The infrared spectrum analysis of the stone components demonstrated a combination of magnesium ammonium phosphate hexahydrate and apatite carbonate, with the former as the main component.

The operation was completed successfully. At 7 days later, the patient was discharged home in good health. He was followed for checking of the recovery status 1 month post-operatively and had no pain or hematuria. Blood examinations including white blood cell and creatinine were normal. A non-contrast computed tomography (CT) scan showed no signs of ureteral dilation or hydronephrosis. Considering that the presence of large BSs might lead to chronic inflammation and squamous cell carcinoma (SCC), the patient was instructed for the risk factors of bladder SCC post-operatively.

**Figure 3:**
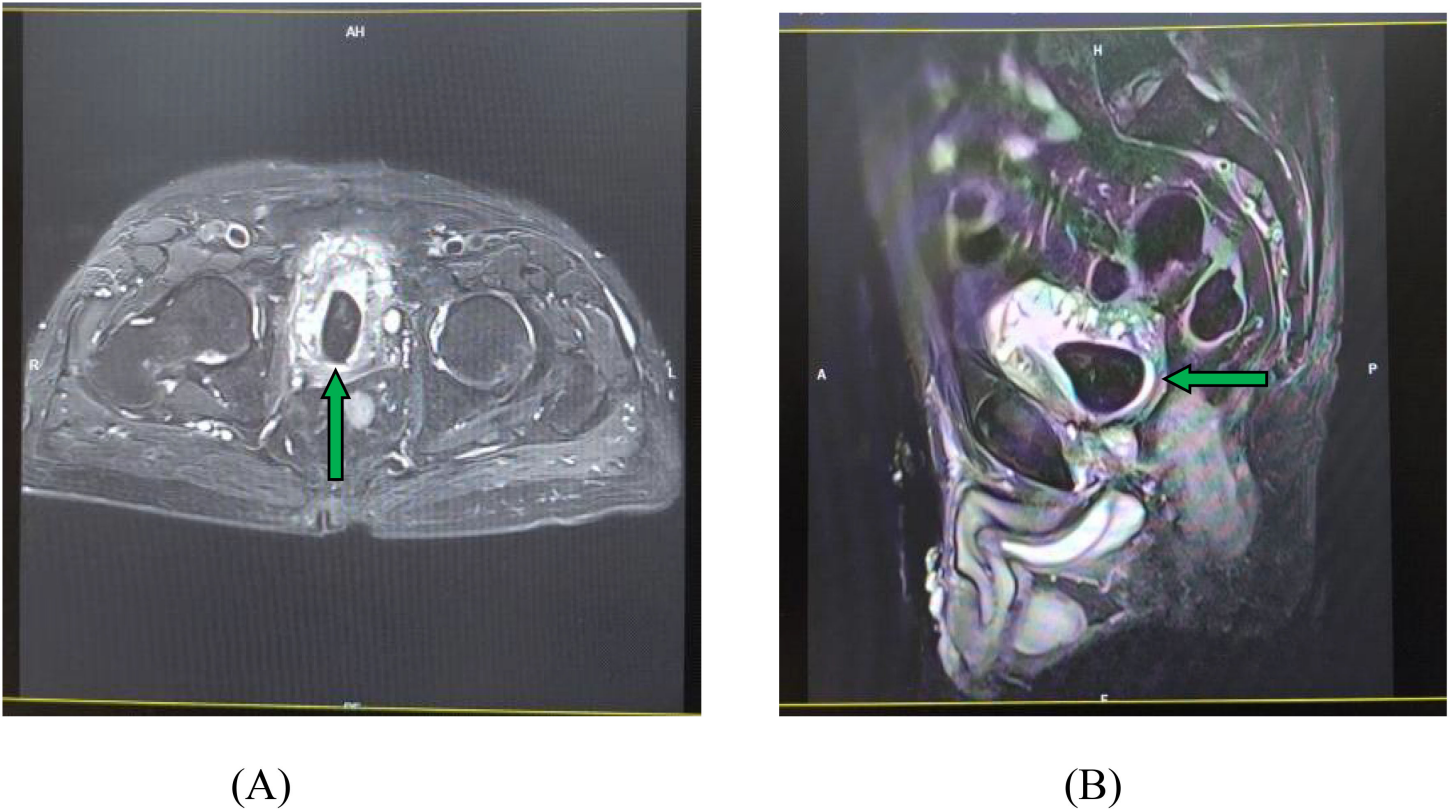
MRI examination of the pelvis showing a large BS. **(A)** Horizontal view. **(B)** Sagittal view.

After a careful re-interpretation of the MRI film with a radiologist, we observed a stone in the bladder, but the stone was missed by the doctors at that tertiary hospital. The MRI film of the pelvis is shown in **Figure 3**.

## Discussion

As one type of urinary stones, BS has been known since the earliest of times. Based on the etiology, BSs are classified as primary, secondary, or migratory (4). Primary BS usually develops in the absence of other urinary tract pathology. These stones are common in pediatric patients residing in developing countries, mainly suffering from malnutrition and poor dietary factors, such as low animal protein diets. Secondary BSs generally occur in the presence of other urinary tract problems, such as benign prostatic hyperplasia, neurogenic bladder dysfunction, bladder diverticula, and bladder augmentation or diversion. Clinically, most BSs are secondary, resulting from upper urinary tract stones, urinary stasis, or recurrent infections (5).

Migratory BS usually refers to the stones caused by the migration of foreign bodies from adjacent organs into the bladder, for example, intra-uterine contraceptive devices perforating the uterus and penetrating into the bladder, previous anti-incontinence tension-free vaginal tape merging into the bladder wall (5), or a bone spur at the time of pelvic fracture and bladder rupture (1). In our case, the cause of BS was a Hem-o-lok clip originating from the previous LRP. Theoretically, the foreign bodies serve as a nidus and generate intravesical stone formation if they stay long enough. Prior case reports in the literature have demonstrated similar cases of bladder stone formation following LRP or robotic-assisted laparoscopic radical prostatectomy (RARP), but the occurrence is rare. A brief summary of the case reports regarding BS caused by migratory Hem-o-lok clip after radical prostatectomy in the literature is listed in **Table 1**. Our case is distinctive for several reasons: First, the stone was exceptionally large (6 × 5 cm, 105 g), which is rare in reported literature. Second, the primary complaint of urinary incontinence was somewhat atypical for a bladder stone and overlapped significantly with symptoms of post-prostatectomy incontinence and neurogenic bladder, which contributed to the diagnostic challenge.

**Table 1:** A brief summary of case reports of BS caused by migratory Hem-o-lok clip after radical prostatectomy.

Author	Age	Clinical presentation	Size of BS	Method of diagnosis	Time after operation	LRP/RARP/retropubic radical prostatectomy	Treatment method
Elizabeth B. et al (6).	53	Persistent bladder spasms and renal colic	1 cm	CT	10 months	LRP	Cystolithopaxy
Shameer D. et al (7).	70	Visible hematuria and worsening LUTS	3.5 cm	CT	11 years	RARP	Cystolithotripsy
Yu-Chen Chen, et al (8).	75	Intermittent gross hematuria	–	CT	5 years	RARP	Laser fragmentation
Jamil Ahmad, et al (9).	76	Total hematuria	1.5 cm	CT	9 years	RARP	Transurethral cystolitholapaxy
Katsumi Kadekawa (10)	75	Painful micturition and gross hematuria	1.5 cm × 2	Radiography (KUB)	3 years	Retropubic radical prostatectomy	Transurethral holmium laser lithotripsy
Our case	75	Incontinence, LUTS, UTI	6 × 5 cm	–	4 years	LRP	Open cystolithotomy

In our case, the previous urethral stricture caused dysuria and urinary stasis, affecting his ability to urinate smoothly, as well as facilitating urinary tract infections. The stone analysis revealed that the main composition was magnesium ammonium phosphate hexahydrate, which is consistent with that of Yung-Shun Juan et al (11). They considered that the infection changed the urine pH values by urea-splitting bacteria; meanwhile, the susceptibility to infection was promoted by the presence of foreign matter, thus leading to the accumulation of crystalline and organic components. Similarly, they also found that foreign body stones were mostly composed of a mixture of struvite and carbonate apatite.

In the literature, a large BS is generally defined as more than 100 g in weight and larger than 4 cm in diameter (2), while some other clinicians defined it as more than 3 cm in size (4). Regardless of which criteria, our case belongs to the scope of large BS. Apparently, it takes a long time for a large BS to form, and for some patients, the initial symptoms of lower urinary tract symptoms (LUTS) could sometimes be overlooked, as these symptoms are non-specific. Because the clinical presentation of BS varies broadly, ranging from asymptomatic to severe symptoms, for example, irritating LUTS, interrupted urination, pain, or even an obstructed renal failure, the diagnosis of BS needs careful determination. However, for patients with typical symptoms, its diagnosis is not very difficult, especially with the use of appropriate imaging examinations. While in some patients, many reasons—asymptomatic, or lower abdominal pain, dysuria, gross hematuria or urinary retention, or misdiagnosis due to unfamiliarity with imaging characteristics on specific modalities like MRI—make the diagnosis challenging, just like what has occurred in our case.

Clinical studies have noted that urinary stones can initially be misdiagnosed due to unclear symptoms (12), so timely and accurate diagnosis is crucial, particularly for old or debilitated patients. Like other urinary stones, imaging examinations are also important in the diagnosis, management, and follow-up of BS. Clinically, several imaging methods have been applied, including serial kidney–ureter–urinary bladder (KUB) X-ray films for radiopaque stones and ultrasonography for radiolucent stones, intravenous pyelography (IVP), CT, and MRI, each having its own advantages and disadvantages (13). In adults, the gold-standard diagnosing method for BS is CT, which scans quickly and clearly and indicates the size, location, and shape of stones with high sensitivity (97%–100%) and specificity (96%–100%). Nevertheless, its chief disadvantage is exposure to ionizing radiation, especially in children (**Table 2**).

**Table 2:** Comparison of commonly used imaging methods for bladder stone diagnosis.

Imaging method	Advantages	Disadvantages
KUB (X-ray)	• Quick and inexpensive • Excellent for radio-opaque stones (e.g., calcium) • Good for tracking stone passage	• Misses radiolucent stones (e.g., uric acid) • Bowel gas/bones can hide stones • No information about kidney function
Ultrasonography	• No radiation (safe for all patients) • Excellent for detecting bladder stones and hydronephrosis • Readily available and portable	• Operator-dependent • Can miss small or ureteral stones • Difficult in obese patients
CT scan (non-contrast)	• Highest accuracy for all stone types/sizes • Fast and not operator-dependent. • Can diagnose other abdominal pains	• High radiation dose • More expensive • Not for pregnant women and children

MRI is a non-radiation imaging modality with high resolution and excellent soft tissue contrast (13). It is well known that in urology, MRI plays important roles in the diagnosis of the location and size of tumors, especially in PCa. MRI can further be used to evaluate the aggressiveness of cancer as well as complications such as abscess or fistula. However, MRI is uncommonly used in urinary stones; thus, some doctors might be unfamiliar with it. Visualization of stones on MRI depends on the contrast between a low signal of the stone and the surrounding structures, but this can only be achieved with few sequences, mainly because the relative absence of protons within the stone makes them incapable of generating a signal on MRI (14). In our case, the large stone, occupying much of the bladder, presented as a well-defined, low-signal intensity mass on T2-weighted images (**Figure 3**). The surrounding high-signal urine created a contrast that, upon retrospective review, was evident. The initial misdiagnosis likely resulted from a combination of factors: the primary clinical suspicion being cancer recurrence or neurogenic bladder, leading to cognitive bias; a lack of specific focus to evaluate the stones; and potentially insufficient experience among both urologists and radiologists in interpreting the stone findings on MRI. Moreover, MRI is more expensive and time-consuming.

As to our case, we thought that he underwent MRI examination at other tertiary hospitals because the urologist was concerned about the relapse of PCa. There was no recurrence of the cancer, but perhaps with few experiences in stone diagnosis with MRI or lack of focus on the stone, the BS was misdiagnosed. Furthermore, this case is particularly interesting because a large stone occupied most of the bladder cavity resulting in bilateral ureteral hydrocele, which disappeared on CT imaging at 1 month postoperatively.

Compared with traditional open radical prostatectomy, LRP has the advantages of a smaller incision, less bleeding, and rapid recovery as well as clearly revealing the anatomy during operation. Meanwhile, as a non-absorbable polymer-clip, Hem-o-lok clip was introduced into laparoscopic procedures to ensure adequate hemostasis and close tissue structures (7) and to control the lateral pedicles in LRP. However, with the increment of surgical volumes and accumulation of clinical experiences, more complications specifically related to Hem-o-lok migration have been identified (15). Of them, the formation of BS after LRP was considered to result from several causes, namely: (1) too tight suture during the intraoperative anastomosis, leading to anastomotic stenosis and secondary bladder stone, (2) postoperative anastomotic scar formation and stenosis, resulting in obstruction and secondary stones; and (3) intravesical foreign body-induced stones, such as sutures or Hem-o-lok. We considered that in our case, the stone was formed by the migration of a Hem-o-lok into the bladder. The possible underlying mechanism of clip migration might be as follows: (1) intraoperatively, improper placement of Hem-o-lok or too near the anastomosis: slow migration and erosion of the clip probably caused bladder stone formation, and it has been suggested that the use of Hem-o-lok clips close to the anastomosis should be omitted. Rassweiler et al. reported that based on this experience, in 2,200 consecutive radical prostatectomy operations, no clip migration was found (8); (1) intraoperatively excessive application of Hem-o-lok clips: researchers found that the incidence of migration was significantly increased when more than four Hem-o-lok clips were applied around the anastomosis; (3) postoperatively, the anastomosis failed to be closed completely within a short period; or (4) loosening of anastomosing sutures, leading to the incorporation of clips into the anastomosis between the thin urethral–bladder anastomotic plate (16), or true migration to the bladder neck owing to scar formation and fibrosis.

In the literature, researchers have proposed to classify clip migrations into three types (15): type I led to obstructive LUTS 2–8 months after prostatectomy, type II caused stone formation, gross hematuria, or bladder spasm, and in type III, the patients expulsed the clip several weeks postoperatively. Based on this criterion, our case belonged to type II.

We considered that several measures could be taken to preclude such misdiagnosis in clinical scenario, on the basis of the lessons learned from our case and the literature reviewed. (1) Understanding the characteristics of BS on MRI examination and being aware mean that the diagnosis of BS formed by foreign bodies requires multiple imaging methods to be confirmed (12). Whenever the diagnosis is doubtful, the underlying reasons must be identified so as to ensure the absolute accuracy of the diagnosis. (2) Recognizing the importance of such complications, during LRP, cautions should be taken when placing clips near the anastomosis, or try not to use clips, while only utilizing sutures and knotting to finish anastomosis. (3) All free-floating clips should also be removed out of the surgical field. (4) An extraordinarily large stone is very rare, so it might be misdiagnosed as pelvic tumor and mislead the treatment. (5) For patients with a previous history of urethral stricture, utmost care must be taken to ensure good micturition status postoperatively, especially when complicated with infection. In addition, much attention should be paid to patients with long-duration BS accompanied by chronic infections for the risk of bladder cancer. Biopsy is recommended if necessary.

## Patient perspective

The patient was very satisfied with the therapeutic result and had provided written consent for the publication of this paper.

## Data Availability

The raw data supporting the conclusions of this article will be made available by the authors, without undue reservation.
